# Entropy-Based Methods for Motor Fault Detection: A Review

**DOI:** 10.3390/e26040299

**Published:** 2024-03-28

**Authors:** Sarahi Aguayo-Tapia, Gerardo Avalos-Almazan, Jose de Jesus Rangel-Magdaleno

**Affiliations:** Digital Systems Group, National Institute of Astrophysics, Optics and Electronics, Puebla 72840, Mexico; sarahi.aguayo@inaoe.mx (S.A.-T.); gerardo.avalos@inaoe.mx (G.A.-A.)

**Keywords:** entropy, motor fault detection, artificial-intelligence-based classifiers, feature vectors

## Abstract

In the signal analysis context, the entropy concept can characterize signal properties for detecting anomalies or non-representative behaviors in fiscal systems. In motor fault detection theory, entropy can measure disorder or uncertainty, aiding in detecting and classifying faults or abnormal operation conditions. This is especially relevant in industrial processes, where early motor fault detection can prevent progressive damage, operational interruptions, or potentially dangerous situations. The study of motor fault detection based on entropy theory holds significant academic relevance too, effectively bridging theoretical frameworks with industrial exigencies. As industrial sectors progress, applying entropy-based methodologies becomes indispensable for ensuring machinery integrity based on control and monitoring systems. This academic endeavor enhances the understanding of signal processing methodologies and accelerates progress in artificial intelligence and other modern knowledge areas. A wide variety of entropy-based methods have been employed for motor fault detection. This process involves assessing the complexity of measured signals from electrical motors, such as vibrations or stator currents, to form feature vectors. These vectors are then fed into artificial-intelligence-based classifiers to distinguish between healthy and faulty motor signals. This paper discusses some recent references to entropy methods and a summary of the most relevant results reported for fault detection over the last 10 years.

## 1. Introduction

Recently, the pursuit of more reliable and accurate techniques for motor fault detection has increased, driving the critical role that electric machines play in various modern industrial applications. Entropy-based methods have gained significant attention among the many emerging methodologies due to their unique ability to capture complex system behaviors and anomalies based on mathematical algorithms.

Entropy, which is a foundational concept that was introduced by Rudolf Julious Emanuel Clausius, has been used as a fundamental tool in signal analysis by assessing the variability and sparsity of signals in different knowledge areas. This pioneering work laid the groundwork for new studies about entropy forms, like information entropy [1], fuzzy entropy [2], and sample entropy [3], which have become useful tools in fault diagnosis methodologies. In recent studies, there has been a growing emphasis on the application of entropy-based methodologies for motor fault detection; for instance, in [4], a feature extraction approach based on entropy was undertaken, where this paper introduced the “weighted multi-scale fluctuation-based dispersion entropy (wtMFDE)” method. Designed for condition monitoring in planetary gearboxes (PGB), wtMFDE harnesses the intricacies of entropy to discern fault signatures from mixed noisy signals. This entropy-based technique seamlessly integrates with adaptive and non-adaptive signal processing methodologies, positioning it ahead of the previously established multi-scale fluctuation-based dispersion entropy (MSFDE) method. When evaluated alongside advanced classifiers, such as multilayer perceptron (MLP), the wtMFDE approach capitalizes on entropy’s power, achieving an unparalleled 100% classification accuracy for specific fault types, as exemplified by sun chipping.

In [5], a fault diagnosis method for rolling bearings leveraging entropy-based techniques is presented. Ensemble empirical mode decomposition (EEMD) initially dissects training samples, with dispersion entropy (DE) quantifying their features. Principal component analysis (PCA) further refines these features, and the Gath–Geva (GG) clustering method categorizes them. When tested against various data sets, including the Case Western Reserve University (CWRU) data set, the method demonstrated its robustness, particularly with DE’s superior stability over other entropy measures and GG’s efficacy in clear sample categorization.

In [6], a method to detect sparking faults in DC motors using stray flux signals is proposed. It employs spectral entropy for signal analysis and introduces a severity indicator based on Mel frequency cepstral coefficients. Evaluations under various motor conditions highlight the method’s consistent effectiveness, positioning it as a promising tool for integrating into DC motor diagnosis systems.

While entropy-based techniques have enriched our understanding of rotatory machine dynamics, there remains a challenge in effectively capturing temporal details. To overcome these temporal limitations, authors have developed multi-scale and multi-modal techniques in order to obtain reliable results [7,8,9].

## 2. Entropy Methods

### 2.1. Shannon Entropy

The first concept of entropy was introduced by Shannon in order to calculate the irregularity and self-similarity of signals. The Shannon entropy H(x) of a random signal *x* with *n* possible outcomes is defined by

(1) $$H\left(x\right)=-\sum_{i=1}^{n}p\left(x_{i}\right)log_{2}\left(p\left(x_{i}\right)\right)$$

where $p(x_{i})$ is the probability density function of the signal $x_{i}$ [10,11].

Shannon entropy can be used to measure a time series’s complexity. By definition, Shannon entropy should be a monotonic increasing function and a continuous function. Lastly, if the probability can be divided into the sum of individual values, so should the Shannon entropy.

Thanks to its characteristics, Shannon entropy is a popular method, not only for fault detection but also for other applications, such as for the analysis of biological signals [12], computational applications [13], and environmental data [14].

#### Reported Works That Used Shannon Entropy

The reported works that used Shannon entropy for fault detection are mostly devoted to analyzing vibration signals. Some of the most relevant works are listed in Table 1, where the methods, type of signals, type of faults, and accuracy of the classification are detailed. Notice that half of these works are proposed to detect bearing faults: inner race (IR), outer race (OR), and ball.

### 2.2. Approximate Entropy

Approximate entropy measures the probability of occurrence of a new pattern based on the observation of the embedding dimension *m* and the similarity coefficient *r*. ApEn is a scale-invariant indicator, given that it relies on the similarity coefficient, which is an equivalent of a standard deviation of a time series.

**Table 1:** Motor fault detection using Shannon entropy.

Year and Author	Methods	Type of Signal (Database)	Type of Fault	Reported Accuracy
2014. Hojat Heidari Bafroui, et al. [15]	Continuous wavelet transform + Shannon entropy + feed-forward MLP	Vibrations (Amirkabir University of Technology)	Gearbox: chipped and worn	94.13–97.21%
2016. David Camarena-Martinez, et al. [11]	K-means cluster + Shannon entropy	Current (own)	1/2 BRB, 1 BRB, and 2 BRBs	95–100%
2017. Shaojiang Dong, et al. [16]	Local mean decomposition + Shannon entropy + fuzzy f-means flustering	Vibrations (CWRU and own)	IR, OR, and ball damage	95%
2022. Yongbo Li, et al. [17]	Local mean decomposition + Shannon entropy + fuzzy f-means flustering	Vibrations (CWRU and own)	IR, OR, and ball damage	95%

ApEn can be defined as follows [10,18]:

(2) $$ApEn=\phi^{m}\left(r\right)-\phi^{m+1}\left(r\right)$$

where $\phi^{m}\left(r\right)$ is the mean value of the logarithm pattern mean count and *r* is the similarity coefficient; on the other hand, $\phi^{m}\left(r\right)$ and $\phi^{\left(m+1\right)}\left(r\right)$ can be calculated with the following expression:

(3) $$\phi^{m}\left(r\right)=\frac{1}{N-m+1}\times \sum_{i=1}^{N-m+1}lnc_{i}^{m}\left(r\right)$$

where $c_{i}^{m}\left(r\right)$ can be defined as follows:

(4) $$c_{i}^{m}\left(r\right)=\frac{n}{N-m+1};i,j=1,2,...,N-m+1,i\neq j$$

Previous studies demonstrated the advantages of the ApEn, such as its insensitivity to inference and noise, its suitability for random and certain signals, and its stable estimation without requiring large amounts of data.

ApEn is also employed for analyzing short data sets [19], computational applications [20], and brain signals [21,22,23,24].

#### Reported Works That Used ApEn

ApEn emerged as an improvement of Shannon entropy, and its use in the fault detection area has been devoted mainly to analyzing vibration signals. In Table 2, some of the relevant works that used ApEn are listed. ApEn-improved methods, like refined composite multi-scale approximate entropy (RCMSAE), are commonly employed together with methods like empirical mode decomposition (EMD) and probabilistic neural network (PNN).

### 2.3. Permutation Entropy

Permutation entropy (PE) considers a signal’s non-linear behavior and describes the time series’s complexity by making a phase space reconstruction. PE only requires the order of the amplitude of the signal. In this regard, this type of entropy has a faster calculation time than others.

**Table 2:** Motor fault detection using ApEn.

Year and Author	Methods	Type of Signal (Database)	Type of Fault	Reported Accuracy
2007. Ruqiang Yan, et al. [25]	ApEn	Vibrations (own)	Structural bearing damage	Not reported
2013. ShuanFeng Zhao, et al. [26]	EMD + ApEn	Vibrations (own)	Bearing: spall-like faults	Not reported
2016. Diego Luchesi Sampaio, et al. [18]	ApEn	Vibrations (own)	Cracked shaft and misalignment	Not reported
2017. Xueli An, et al. [27]	ApEn + k-nearest neighbor + adaptive local iterative filtering	Vibrations (own)	IR, OR, and ball bearing fault	100%
2021. Jianpeng Ma, et al. [28]	RCMSAE + improved coyote optimization-PNN	Vibrations (CWRU and own)	IR, OR (CWRU and own), and ball bearing faults	94.9% (CWRU) and 93.9% (own)

PE can be expressed in terms of the relative frequency p(π) for each permutation π as follows [10,29]:

(5) $$PE=-\sum p\left(\pi \right)log_{2}p\left(\pi \right)$$

(6) $$p\left(\pi \right)=\frac{\mathrm{num}\{X_{i}^{m}\mathrm{has}\;\mathrm{type}\;\pi ,i|1,2,...,N-m+1\}]}{N-m+1}$$

PE is an adequate indicator of the complexity of signals from nonlinear processes; furthermore, PE’s advantages have been highlighted in other works, such as its high calculation efficiency, its robust ability against noise, and its good complexity estimation [10].

Some other applications of PE include the analysis of electroencephalographic signals [30,31,32] and financial time series [33,34].

#### Reported Works That Used PE

Permutation-entropy-based methods for fault detection are listed in Table 3. Notice that all of these works are devoted to the detection of bearing faults by using vibration signals, as is common in most of the works that use PE [35,36,37,38,39,40].

Together with PE, methods for signals processing and fault classification are used, such as continuous wavelet coefficient (CWC), ensemble empirical mode decomposition (EEMD), support vector machine (SVM), adaptive neuro-fuzzy inference system (ANFIS), flexible analytical wavelet transform (FAWT), composite multi-scale permutation entropy (CMSWPE), grey wolf optimizer (GWO) SVM, composite multi-scale permutation entropy (CMSPE), reverse cognitive fruit fly optimization algorithm (RCFOA), particle swarm optimization (PSO), improved multi-scale permutation entropy (IMSPE), and extreme learning machine (ELM). Moreover, not only is PE employed but also improved methods, such as multi-scale permutation entropy (MSPE), generalized composite multi-scale permutation entropy (GCMSPE), and time-shift multi-scale weighted permutation entropy (TSMSWPE) [41].

### 2.4. Sample Entropy

Sample entropy (SE) measures the irregularity of a signal independent of the similarity coefficient *r* and the embedding dimension *m*.

**Table 3:** Motor fault detection using PE.

Year and Author	Methods	Type of Signal (Database)	Type of Fault	Reported Accuracy
2013. Shuen-De Wu, et al. [29]	MSE, MSPE, MBSE, and MSRMS + SVM	Vibrations (CWRU)	IR, OR, and ball bearing damage	96.01–99.79%
2014. Vakharia, et al. [42]	CWC + PE + SVM	Vibrations (CWRU)	IR, OR, and ball bearing damage	97.5%
2016. Yongbo Li, et al. [43]	Local mean decomposition + MSPE + Laplacian score + improved SVM based on binary tree	Vibrations (CWRU)	IR, OR, and ball bearing damage	97.5%
2017. Jinde Zheng, et al. [44]	GCMSPE + Laplacian score + PSO-based SVM	Vibrations (CWRU and own)	IR, OR (CWRU and own), and ball bearing damage (CWR)	88.89–100% (CWR) and 96.67–100% (own)
2018. Moshen Kuai, et al. [45]	Complete EEMD with adaptative noise + PE + ANFIS	Vibrations (own)	Gear faults: broken, one missing tooth, and tooth root crack	80–100%
2019. Wenhua Du, et al. [46]	SOF logic classifier + MSPE + LDA	Vibrations (CWRU and own)	IR, OR, and ball damage (CWRU); cracked and peeled bearing (own)	92.66–100% (CWR) and 97.75–99.25% (own)
2019. Jinde Zheng, et al. [47]	CMSWPE + ELM	Vibrations (CWRU and Suzhou University)	IR, OR (CWRU and Suzhou U.), and ball bearing damage (CWR)	90.48–100% (CWR) and 100% (Suzhou U.)
2019. Xiaoming Xue, et al. [48]	PE + VMD + RF	Vibrations (CWRU)	IR, OR, and ball damage (CWRU)	98.44% and 99.09% for different loads
2019. Zhilin Dong, et al. [49]	TSMSWPE + GWO-SVM	Vibrations (CWRU)	IR, OR, and ball damage (CWRU and Soochow University)	100% (CWR) and 93.5–100% (Soochow U.)
2020. Snehsheel Sharma, et al. [50]	PE + FAWT + SVM	Vibrations (CWRU)	IR, OR, and ball damage	95–100%
2020. Cheng He, et al. [51]	CMSPE + RCFOA-ELM + PSO-VMD	Vibrations (CWRU)	IR, OR, and ball damage	97.33–98.67%
2021. Amrinder Singh Minhas, et al. [52]	IMSPE + dominant statistical parameters + extreme gradient boosting	Vibrations (CWRU and own)	IR, OR (CWRU and own), and ball damage (CWRU)	96.6–100% (CWRU) and 96.2–100% (own)
2021. Govind Vashishtha, et al. [53]	ELM + SWD + PE	Vibrations (CWRU and own)	IR, OR (CWRU and own), and ball damage (CWRU)	100%

Consider a signal **S** of data length *N* expressed by $S=\{x_{1},x_{2},...,x_{N}\}$. A pattern is formed by *m* sequential points of the signal **S**; for example, $X_{i}=\left[x_{i},x_{i+1},...,x_{i+m+1}\right]$ would represent the *i*th pattern. Hence, the pattern space **X** is defined as follows:

(7) $$X=\left[\begin{array}{cccc} x_{1} & x_{2} & \cdots & x_{m}\\ x_{2} & x_{3} & \cdots & x_{m+1}\\ \vdots & \vdots & \ddots & \vdots \\ x_{N-m+1} & x_{N-m+2} & \cdots & x_{N} \end{array}\right]$$

SE can be calculated as follows:

(8) $$SE=-ln(\frac{B^{m+1}\left(r\right)}{B^{m}\left(r\right)})$$

where $B^{m}\left(r\right)$ represents the mean value of the pattern mean count; $B^{m}\left(r\right)$ and $B^{m+}\left(r\right)$ are calculated according to the following expression:

(9) $$B^{m}\left(r\right)=\frac{1}{N-m}\frac{1}{N-m+1}\sum_{i=1}^{N-m}\sum_{j=1}^{N-m+1}G\left(d_{ij},r\right)$$

where $d_{ij}=∥X_{i}-X_{j}∥$ and G(·) is the Heaviside function. In the context of SE, the suggestion of use for *r* is to select a value of 0.2 times the standard deviation of the data set [54].

Besides the motor’s fault detection, other applications that rely on SE’s properties are biomedical [55,56], electrical vehicles [57,58], and weather data series [59].

#### Reported Works That Used SE

In the following Table 4, a summary of some of the most relevant works that utilized SE and improved methods, such as generalized refined composite multi-scale sample entropy (GRCMSSE) for motor fault detection, is presented. Most of them aim to detect bearing faults, but two of the cited works propose the detection of gear and impeller faults.

**Table 4:** Motor fault detection using SE.

Year and Author	Methods	Type of Signal (Database)	Type of Fault	Reported Accuracy
2015. Minghong Han, et al. [60]	Local mean decomposition + SE + SVM	Vibrations (CWRU)	IR, OR, and ball bearing damage	100%
2017. Qing Ni, et al. [61]	SE, root-mean-square value, crest, and kurtosis	Vibrations (Lu Nan wind farm)	IR bearing fault	Not reported
2019. Yongbo Li, et al. [62]	MSSE + Vold–Kalman filter + least squares SVM	Vibrations (UESTC)	Gear fault: cracked tooth and distributed wear	100%
2019. Zhaoyi Guan, et al. [63]	EMD + SE + deep belief network	Vibrations (own)	Structural faults	99–100%
2020. Zhenya Wang, et al. [64]	GRCMSSE + S-isomap + Grasshopper optimization algorithm-SVM	Vibrations (Drivetrain diagnostics simulator)	IR, OR, and ball bearing faults	100%

### 2.5. Fuzzy Entropy

Fuzzy entropy (FE) emerged as an improvement of the sample entropy because FE uses a Gaussian function for measuring the similarity between two time series instead of the Heaviside function that SE uses.

Given a signal u(i),i=1,2,...,N of *N* samples, a vector set $\left\{X_{i}^{m},i=1,2,...,N-m+1\right\}$ is formed. Each vector has *m* sequential elements from the signal u(i) in the form of

(10) $$X_{m}^{i}=\left\{u\left(i\right),u\left(i+1\right),...,u\left(i+m-1\right)\right\}-u_{o}\left(i\right)$$

where $u_{o}\left(i\right)$ represents the average of the vector $X_{i}^{m}$.

Then, the similarity FE for a time series is defined as follows:

(11) $$D_{ij}^{m}=\mu \left(d_{ij}^{m},n,r\right)=e^{-ln2{\left(d_{ij}^{m}/r\right)}^{n}}$$

where $d_{ij}^{m}$ is the distance between $X_{i}^{m}$ and $X_{j}^{m}$, *r* represents the similarity tolerance, and $\mu (d_{ij}^{m},n,r)$ is a fuzzy function.

On the other hand, the function $\varphi^{m}\left(n,r\right)$ is expressed as

(12) $$\varphi^{m}\left(n,r\right)=\frac{1}{N-m}\sum_{i-1}^{N-m}\left(\frac{1}{N-m-1}\sum_{j=1,j\neq i}^{N-m}D_{ij}^{m}\right)$$

Finally, FE can be defined as follows [10]:

(13) $$FE\left(m,n,r,N\right)=ln\varphi^{m}\left(n,r\right)-ln\varphi^{m+1}\left(n,r\right)$$

FE considers the ambiguous uncertainties from the highly irregular time series, making it insensitive to background noise.

Fuzzy entropy has been applied in different fields, like image processing [65,66], the analysis of biomedical signals [67,68,69,70], and decision making [31,71,72].

#### Reported Works That Used FE

In Table 5, a summary of some of the most relevant works that utilized FE for motor fault detection is presented. Most of them aim to detect bearing faults, but two of the cited works propose the detection of gear and impeller faults.

Some of the improved methods based on FE are multi-scale fuzzy entropy (MSFE), refined composite multi-scale fuzzy entropy (RCMSFE), generalized composite multi-scale fuzzy entropy (GCMSFE), multi-scale refined composite standard deviation fuzzy entropy (MSRCSDFE), and multivariable multi-scale fuzzy distribution entropy (MMSFDE). Although these methods extend the scope of FE by adding, for example, the multi-scale or the generalized analysis, all of them are still driven by FE principles [73,74,75,76,77,78,79,80,81,82,83].

**Table 5:** Motor fault detection using FE.

Year and Author	Methods	Type of Signal (Database)	Type of Fault	Reported Accuracy
2016. Huimin Zhao, et al. [84]	EEMD + MSFE + SVM	Vibrations (CWRU)	IR, OR, and ball bearing damage	95–100%
2018. Wu Deng, et al. [85]	EWT + FE + SVM	Vibrations (simulated signals)	IR, OR, and ball bearing damage	90–100%
2018. Jinde Zheng, et al. [86]	Sigmoid-based RCMSFE + t-SNE + VPMCD	Vibrations (CWRU)	IR, OR, and ball bearing damage	100%
2018. Yu Wei, et al. [74]	Intrinsic characteristic-scale decomposition + GCMSFE + Laplacian score + PSO-SVM	Vibrations (Harbin Intitute of Technology and CWRU)	IR, OR (Harbin I.T. and CWR), and impeller faults (Harbin I.T.)	98.13–100% (Harbin I.T.) and 100& (CWRU)
2019. Amrinder Singh Minhas, et al. [87]	MSRCSDFE + EEMD	Vibrations (own)	IR and OR	92.77–100%
2021. Xu Chen, et al. [88]	RCMSFE + out-of-sample embedding + MPA-SVM	Vibrations (CWRU and own)	IR and OR	100%
2021. Yanli Ma, et al. [28]	MMSFDE + Fisher score + SVM	Vibrations (Hunan University and own)	Drive gear (case 1) and bearing + gear fault (case 2)	97.71–100% (case 1) and 92.5–99.5% (case 2)
2022. Yongbo Li, et al. [17]	SFE and MSFE	Vibrations (ADVC Laboratory and Paderborn University)	OR faults: sharp trench, drilling, pitting (ADVC), and rubbing (Paderborn U.)	99.88% (ADVC) and 99.3% (Paderborn U.)

### 2.6. Energy Entropy

Energy entropy (EE) estimates a signal’s complexity based on its intrinsic mode functions (IMFs). Its calculation starts with the energy of the *i*th IMF as follows:

(14) $$E_{i}=\sum_{j=1}^{m}{\left|c_{ij}\right|}^{2}$$

where *m* is the length of the IMF. Then, the total energy of the *n* IMFs is given by

(15) $$E=\sum_{i=1}^{n}E_{i}$$

Finally, the energy entropy $H_{en}$ of the signal is calculated based on the following expression:

(16) $$H_{en}=-\sum_{j=1}^{n}p_{i}log\left(p_{i}\right)$$

where $p_{i}=E_{i}/E$ represents the percentage of the *i*th IMF relative to the total energy entropy [10].

The energy entropy provides very good results when analyzing non-stationary and nonlinear complex signals; for example, if a fault in the motor provokes a change in the signal’s frequency, the energy distribution will change. Hence, energy entropy can be used to effectively portray the signal’s characteristics [89].

Other fields besides fault detection where the EE has been applied are milling chatter detection [90], computational chemistry [91], and thermomechanics applications [92].

#### Reported Works That Used EE

Some of the latest relevant works that used energy entropy for fault detection are listed in Table 6. Unlike the previously mentioned methods, by using EE, more types of faults have been detected, such as misalignment, imbalance, and bearing faults. It is also important to recall that one of these works relied on current signals for the analysis [93,94].

**Table 6:** Motor fault detection using EE.

Year and Author	Methods	Type of Signal (Database)	Type of Fault	Reported Accuracy
2017. Yancai Xiao, et al. [89]	IEMD energy entropy + PSO + SVM	Vibrations (own)	Parallel, angle, and comprehensive misalignment	98.913%
2017. Yancai Xiao, et al. [93]	Dual-tree complex wavelet transform + EE + PSO	Current (simulation)	Parallel, angle, and comprehensive misalignment	96%
2018. Bin Pang, et al. [95]	CFBEE + improved singular spectrum decomposition + Hilbert transform + SVM	Vibrations (own)	Local rubbing, oil film whirl, and imbalance fault	100%
2021. Shuzhi Gao, et al. [96]	IEE + triangulation of amplitude attenuation + correlation analysis	Vibrations (own)	IR, OR, and ball bearing damage	91–99.67%

Improved methods for EE are also proposed for fault detection, such as characteristic frequency band energy entropy (CFBEE) and improved energy entropy (IEE).

### 2.7. Dispersion Entropy

The dispersion entropy (DE) of a signal *x* of *n* samples can be calculated with the following steps [97,98]:

First, the signal *x* is normalized between 0 and 1. To do so, a sigmoid function is usually employed for this mapping. Some works have reported using normal cumulative distribution functions (NCDF) for this step [97,98]. Hence, the time series *y* is obtained from the NCDF of the signal *x*, which is defined as follows:

(17) $$y_{i}=\frac{1}{\sigma \sqrt{2\pi }}\int_{-\infty }^{x_{i}}e^{\frac{-{\left(t-\mu \right)}^{2}}{2\sigma^{2}}}dt$$

where σ represents the standard deviation and μ is the mean of the signal *x*.

The second step consists of mapping the time series *y* to *c* classes by multiplying $y_{i}$ by *c*, then adding 0.5 and rounding to the nearest integer, as follows:

(18) $$z_{i}^{c}=\mathrm{round}\left(c\times y_{i}+0.5\right)$$

where $z_{i}^{c}$ represents the *i*th term of the classified time series $z^{c}$.

In the third step, the time series $z_{j}^{m,c}$ is constructed based on the embedding dimension *m* and the time delay *d*:

(19) $$z_{j}^{m,c}=\left\{z_{j}^{c},z_{j+d}^{c},...,z_{j+(m-1)d}^{c}\right\}j=1,2,..,N-\left(m-1\right)d$$

Then, $z_{j}^{m,c}$ is mapped into a dispersion pattern $\pi_{v0v1...v_{m-1}}$:

(20) $$z_{i}^{c}=v0,z_{i+d}^{c}=v1,z_{i+2d}^{c}=v2,...,z_{i+(m-1)d}^{c}=v_{m-1}$$

Here, the number of feasible dispersion patterns is $c^{m}$ given that each $z_{j}^{m,c}$ is conformed by *m* elements, which can be an integer from to *c*.

The fourth step corresponds to the calculation of the relative frequency of each dispersion pattern $\pi_{v0v1...v_{m-1}}$, which is given by

(21) $$p\left(\pi_{v0v1...v_{m-1}}\right)=\frac{\mathrm{num}\{j|j\leq N-\left(m-1\right)d,z_{j}^{m,c}\mathrm{has}\;\mathrm{type}\;\pi_{v0v1...v_{m-1}}\}}{N-(m-1)d}$$

Finally, the DE is calculated as follows:

(22) $$DE\left(x,m,c,d\right)=-\sum_{\pi =1}^{c^{m}}p\left(\pi_{v0v1...v_{m-1}}\right)\times ln\left(p\left(\pi_{v0v1...v_{m-1}}\right)\right)$$

where *m* represents the embedding dimension, *c* is the number of classes, and *d* is the time delay.

Some works prefer to express the DE in its normalized form, which is given by

(23) $$NDE\left(x,m,c,d\right)=\frac{DE(x,m,c,d)}{ln(c^{m})}$$

The advantages of DE have been used for other applications, such as the analysis of biomedical signals [70] and image processing [99].

#### Reported Works That Used DE

In Table 7, relevant works that used DE for motor fault detection are listed. Notice that DE has become popular, especially during the last few years; authors rely on this method due to its high stability.

Some of the improved methods based on DE are hierarchical symbolic dynamic entropy (HSDE), improved multi-scale dispersion entropy (IMSDE), refined composite multi-scale dispersion entropy (RCMSDE), weighted refined composite multi-scale dispersion entropy (WRCMSDE), time-shift multi-scale dispersion entropy (TSMSDE), multi-scale dispersion entropy (MSDE), and stacking modified composite multi-scale dispersion entropy (SMCMSDE).

**Table 7:** Motor fault detection using DE.

Year and Author	Methods	Type of Signal (Database)	Type of Fault	Reported Accuracy
2018. Mostafa Rostaghi, et al. [97]	HSDE	Vibrations (CWRU, University of Tabriz)	IR, OR, ball bearing faults (CWRU), and medium worn and broken teeth of a spur gear of the gearbox (U. of Tabriz)	Not reported
2018. Xiaoan Yan, et al. [98]	IMSDE + mRMR + ELM	Vibration (CWRU)	IR, OR, and ball bearing faults	+98%
2019. Weibo Zhang, et al. [100]	RCMSDE + fast EEMD + mRMR + random forest classifier	Vibration (CWRU)	IR, OR, and ball bearing faults	96.6–100%
2020. Amrinder Singh Minhas, et al. [101]	Complementary EEMD + WRCMSDE, WRCMSFE, WRCMSPE + SVM	Vibration (CWRU and own) and acoustics (own)	IR, OR (CWRU and own), and ball bearing faults (CWRU)	70–100%
2020. Kaixuan Shao, et al. [102]	VMD + TSMSDE + SVM + vibrational Harris hawks optimization	Vibration (CWRU and Cincinnati IMS)	IR, OR, and ball bearing faults	96.56–98.81% (CWRU) and 79–100% (IMS)
2021. Snehsheel Sharma, et al. [7]	Multi-scale fluctuation based DE + local mean decomposition + SVM	Vibration (CWRU)	IR, OR, and ball bearing faults	98–100%
2021. Xiong Zhang, et al. [5]	EEMD + MSDE + PCA + Gath–Gera clustering method	Vibration (CWRU, QPZZ-II, and Cincinnati IMS)	OR (all), IR, and ball bearing faults (CWRU and QPZZ-II)	100%
2021. Hongchuang Tan, et al. [103]	SMCMSDE + equilibrium optimizer-SVM + complete EEMD with adaptative noise	Vibration (CWRU and own)	IR, OR, and ball bearing fault	99.75% (CWRU) and 99.9% (own)
2021. Qiang Xue, et al. [104]	HDE + joint approximate diagonalization of eigenmatrices	Vibration (CWRU and own)	IR, OR, and ball bearing faults	100%
2021. Fuming Zhou, et al. [105]	MHMSFDE + multi-cluster feature selection + GWO based kernel ELM	Vibration (CWRU and QPZZ-II)	IR, OR, ball bearing faults (CWRU), pinion wear, gearwheel pitting, gearwheel tooth breaking, and gearwheel pitting + pinion wear (QPZZ-II)	100% (CWRU) and 98.5–99.24% (QPZZ-II)

### 2.8. Multi-Scale Entropy

The multi-scale version of any type of entropy method consists of the calculation of the entropy at different scales. To this end, a coarse-grained data sequence $y_{j}^{\left(s\right)}$ should be obtained by a coarse-grained process of the original signal *x*. Then, $y_{j}^{\left(s\right)}$ can be expressed as follows [64]:

(24) $$y_{j}^{\left(s\right)}=\frac{1}{s}\sum_{i=(j-1)s+1}^{js}x_{i};j=1,2,...,\frac{N}{s}$$

where *s* represents a scale factor. Therefore, the signal *x* is transformed into a coarse grain sequence of length N/s.

The multi-scale entropy (MSE) accuracy is constrained by the single-scale method; however, it is usually preferred over the one-scale analysis because it provides more information despite the increase in the calculation time.

The type of applications where the MSE can be used are as vast as the applications of each single-scale method, such as the analysis of time series [106,107]; biological signals, such as heartbeats and encephalographics [108,109,110]; image processing [111]; and hydrologic applications [112]. There are some interesting works on improvements around the MSE, as presented in [113], where the authors successfully diagnosed gearbox and milling tool faults. The method utilizes a novel technique that combines MPE with contrastive learning (LE), yielding results that improve the accuracy of traditional entropy-based methods.

Finally, in Table 8, a summary of each method’s advantages and disadvantages is presented to provide a wider panorama of its characteristics.

### 2.9. Practical Example: Applied Entropy Methods for Broken Bar Detection

**Figure 1:**
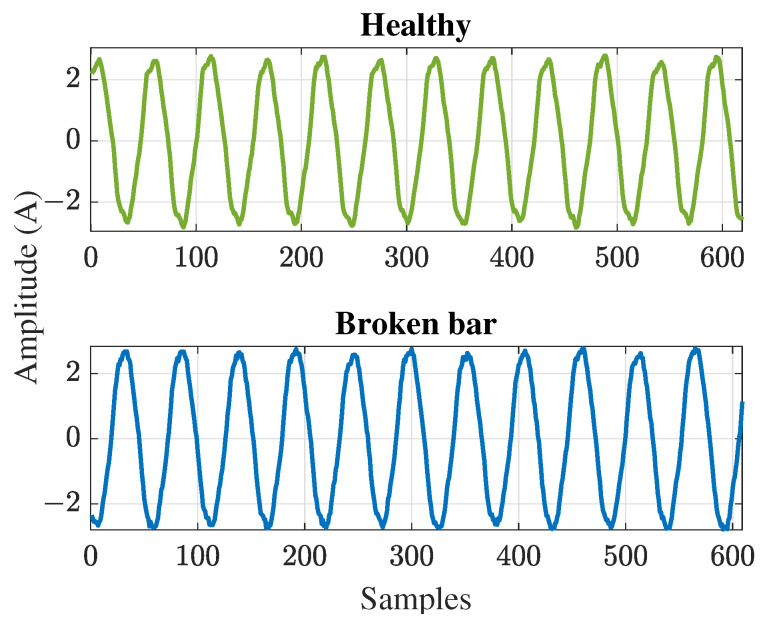
Current signals from the two conditions of a motor: healthy and one broken bar.

To provide an example of the use of different entropy methods and their effects on the classification accuracy, an implementation of three of the methods presented in this paper was conducted: Shannon entropy, approximate entropy, and energy entropy. These three methods were applied to the same signals from a motor with a healthy bar (HB) and with a broken bar (BB), without any preprocessing. A set of 50 current signals in the steady state were analyzed, as shown in Figure 1. Further explanation about the experimental setup to acquire these signals can be found in [114], where the authors performed an early broken bar detection. As can be observed from Figure 1, the signals were quite similar; therefore, an entropy method could be helpful to discern between the two conditions of the motor.

**Figure 2:**
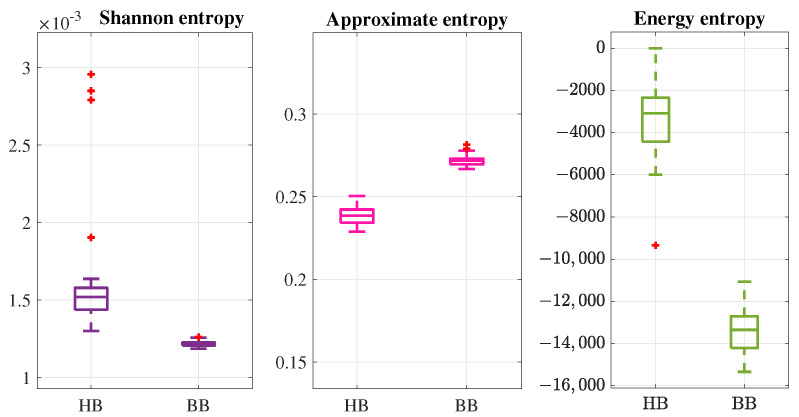
Comparison of three different entropy methods for a practical case of broken bar fault detection.

Results are displayed in Figure 2 comparing the entropy for the two conditions of the motor. Notice that the use of entropy allowed for a separability of data in a similar way that other traditional methods could provide, such as motor current signature analysis. According to the nature of the signal and the aim of the analysis, a certain method of entropy could be more useful than others. For example, energy entropy could be more suitable for this application since it only depends on the intrinsic characteristics of the signal. It is worth noticing from Figure 2 that the separability of data using this type of entropy is better than using Shannon or approximate entropy. Actually, approximate entropy is commonly employed for vibration signals, which are usually more irregular than current signals. Also, according to the characteristics of the phenomenon, certain entropy methods could be discarded; for example, when analyzing a high-frequency phenomenon, dispersion entropy is not adequate.

The selection of the type of entropy is also dependent on its application. A signal with higher separability, such as the comparison between a healthy motor and a motor with a medium level of damage, could be successfully classified with more straightforward methods, such as Shannon entropy, or a faster method, such as permutation entropy. But regarding a more complex analysis, a multi-scale analysis could be necessary.

## 3. The Role of Entropy in the Fault Diagnosis of Electromechanical Systems: Challenges and Advances

As a statistical measure, entropy is capable of quantifying the complexity of signals, which is closely related to the functional status of an electromechanical system. Consequently, entropy emerges as a promising non-parametric tool to extract characteristics from a system. Recently, several studies applied entropy indices for fault diagnosis, detection, and prediction in electric machines. Some of them employed more than one entropy index to obtain a multi-modal analysis. Despite the existence of several entropy-based algorithms for fault detection, most of them are based on Shannon entropy for random or deterministic behavior detection in signals from electric machines. The different forms of entropy employed for fault detection are usually based on the assessment of aleatory and complexity metrics of the signals, and any change in these indices could be related to important changes in the system behavior.

Depending on the nature of the signals, a specific index may be more useful than other; for this reason, it is necessary to apply different entropy metrics in combination with different classification methods, with the aim to cover all the possible faults. The entropy indices described in Section 2.1, Section 2.2, Section 2.3, Section 2.4, Section 2.5, Section 2.6 and Section 2.7 are commonly used for fault detection. Unfortunately, the classical models of these entropy-based indices are only useful for analyzing signals at one level (monoscale analysis), which does not provide the complete feature extraction of the signal.

To overcome the limitations of a monoscale analysis, multiscale-entropy-based methods were proposed, such as the method presented in Section 2.8. Despite their advantages, there exist some problems with this kind of method, like indeterminacy problems and instability for short signals, in addition to its low sensibility for high-frequency systems. Based on these, the main challenge and the current research status on entropy-based methods is a multiresolution analysis, which is needed to obtain indices that entirely describe the dynamics of the signal under study based on all of its oscillatory components [115,116,117,118]. In general, new entropy-based methods aim to provide information about the signal’s state at various levels of oscillation, and thus, better extract the characteristics of the signals under study in order to detect a fault. It is important to mention that the actual trend is the combination of entropy indices with artificial intelligent methods to improve the accuracy of the control systems and fault classification. Another important aspect about entropy based methods is the computational complexity, which allows for online hardware implementations.

However, the advantages of entropy-based methods are evident, in contrast with other methodologies, due the capability of the entropy indices to give information about the dynamics at different abstraction levels of the electromechanic systems. Some of the information aspects provided by entropy indices are systems complexity, stability and regularity, changes detection, resilience to disturbances, hidden patterns and structures, anomalies detection, future events prediction, and model validation, among others. In contrast to other methods, the calculation of entropy indices does not require a large amount of data, nor does it depend on the model and parameters of electric machines.

**Table 8:** Advantages and disadvantages of different entropy methods.

Method	Advantages	Disadvantages
ShanEn	Allows for the assessment of the quantity of information in a signal. It is the basis of the following methods.	Its value only depends on the elements with probability ≠ 0; therefore, some elements could be neglected.
ApEn	Uncertainty estimation regarding future observations based on past observations.	Dependent on the selection of the hyperparameters. Dependent on the length of the signal. Self-similarity feature [87].
SE	Better performance and less sensitivity to data length compared with ApEn	Dependent on selecting the hyperparameters. Similarity criteria dependent on the Heaviside function [50].
FuzzyEn	Better consistency and less dependent on the signal length compared with SE. Reflects the complexity and self-similarity features of a signal in a better way than SE and ApEn.	Dependent on the selection of parameters.
PerEn	High computational speed. Suitable for stationary and non-stationary signals.	Low discrimination capacity given that it does not consider amplitude values.
DE	Faster calculation speed than PerEn. High stability.	Only analyzes the low-frequency part of the signal.
MSE	Analyzes the signal in multiple scales	Efficiency dependent on the single-scale entropy method. Slower method given the entropy calculation within a range of scales.

## 4. Future Trends

Over the years, the use of entropy methods has evolved, with the aim to obtain more accurate and robust results. To this end, improved methods were proposed, such as generalized, multi-scale, composite, hierarchical, and multivariable entropy methods.

Some works also proposed combined methods in order to overcome the drawbacks of using only one type of entropy. But most importantly, entropy methods are usually employed together with signal processing techniques, such as PCA, EMD, and EWT. Artificial-intelligence-based classifications are also commonly used with entropy methods to achieve good classification accuracies when more than one type of fault is being analyzed.

As a summary, some of the trends observed during the elaboration of this work are listed below:

- Most of the entropy methods are applied to vibration signals. This can be attributed to the nature of the signal and the straightforward acquisition. The presence of a fault in a motor usually increases the complexity of the vibration signal, given that it would introduce abnormal components in the spectrum. In this regard, it is expected that vibration analysis remains the preferred type of signal for entropy-based fault detection techniques.
- Bearing fault detection is the type of fault that is mostly covered in entropy-based works. Other faults analyzed with entropy methods are gearbox faults, misalignment, and broken rotor bars, among other less common faults. However, these types of fault represent less than 10% of the work compared with those that analyze bearing faults.
- PE and FE are the most popular methods for motor fault detection. During the last few years, DE has also gained attention. Therefore, it is expected that these would remain the preferred methods, along with their variations, such as composite, weighted, refined, generalized, and multi-variable approaches.
- The development of new entropy-based methods for multiresolution analysis to cover more than one oscillation pattern.
- Multimodal analysis in combination with artificial intelligence techniques for monitoring, control, and multiple fault detection.
- Adaptive entropy-based techniques capable of dynamically adjusting to change the operational conditions of electric motors.
- Emphasis on computational complexity improvements based on algorithmic optimization techniques.
- Hardware implementation of entropy-based methodologies for online monitoring.

It is important to mention that aspects such as algorithmic optimization and hardware implementation are fundamental areas of study. These areas aim to adapt fault detection technology to the emerging trends in electrical systems, particularly in line with the philosophy of smart systems that embrace trends like Industry 4.0 and the Internet of things.

## 5. Conclusions

Different entropy methods were proposed over the years, with some of them aiming to improve the performance of the older ones. In general, the entropy methods are used for extracting characteristics of the motor’s signal to provide a classification that is commonly based on artificial intelligence.

Vibration analysis stands out as the preferred signal type among all the entropy methods reported in this work. In the future, it would be valuable for the state of the art to propose analysis based on other physical variables, such as the current or flux.

In the same regard, the analysis of a wider range of faults would be valuable given that over the years; the focus has been maintained on bearing fault detection.

Fuzzy entropy and dispersion entropy are some of the most reliable methods for entropy-based fault detection thanks to their high stability and reliability, and they are not dependent on the selection of parameters, like the sample entropy and approximate entropy. Permutation entropy is another popular method, and it has shown very good classification accuracies when applied with a classification method like SVM or ELM.

Multi-scale entropy has been preferred in recent years given that it provides more accurate results than a one-scale entropy analysis. Although selecting a multi-scale analysis could have the drawback of a slower calculation, usually, this is not relevant given that the progression of a fault, such as a bearing fault, is rather slow compared with the computation times of the method.

As machinery includes more sophisticated technologies and the demand for uninterrupted services by society increases, it is imperative to find new efficient and accurate mechanisms for fault detection and classification. Entropy-based methods are poised to play a pivotal role in the next generation of monitoring and control systems in conjunction with machine learning methods due to their capability to detect changes in dynamic systems over time.

## Data Availability

Not applicable.

## Abbreviations

The following abbreviations are used in this manuscript:

ANFIS	Adaptive neuro-fuzzy inference system
ApEn	Approximate entropy
CFBEE	Characteristic frequency band energy entropy
CMSPE	Composite multi-scale permutation entropy
CMSWPE	Composite multi-scale weighted permutation entropy
CWC	Continuous wavelet coefficients
CWRU	Case Western Reserve University
DE	Dispersion entropy
EE	Energy entropy
EEMD	Ensemble empirical mode decomposition
ELM	Extreme learning machine
EMD	Empirical mode decomposition
EWT	Empirical wavelet transform
FAWT	Flexible analytical wavelet transform
GCMSFE	Generalized composite multi-scale fuzzy entropy
GCMSPE	Generalized composite multi-scale permutation entropy
GRCMSE	Generalized refined composite multi-scale sample entropy
GCMSSDE	Generalized composite multi-scale symbol dynamic entropy
GCMSWPE	Generalized composite multi-scale weighted permutation entropy
GRCMSSE	Generalized refined composite multiscale sample entropy
GWO	Grey wolf optimizer
HSDE	Hierarchical symbolic dynamic entropy
HDE	Hierarchical dispersion entropy
HSE	Hierarchical sample entropy
HPE	Hierarchical permutation entropy
IEE	Improved energy entropy
IEMD	Improved empirical mode decomposition
IMSDE	Improved multi-scale dispersion entropy
IMSPE	Improved multi-scale permutation entropy
IR	Inner race
ISSD	Improved singular spectrum decomposition
IMS	Intelligence maintenance systems
LDA	Linear discriminant analysis
MBSE	Multi-band spectrum entropy
MHSE	Marginal Hilbert spectrum entropy
MLP	Multi-layer perceptron
WSST	Wavelet semi-soft threshold
MPA	Marine predators algorithm
MSE	Multi-scale entropy
MSDE	Multi-scale dispersion entropy
MSFDE	Multi-scale fluctuation-based dispersion entropy
MSFE	Multi-scale fuzzy entropy
MSSFE	Multi-scale symbolic fuzzy entropy
MMSFDE	Multivariable multi-scale fuzzy distribution entropy
MSPE	Multi-scale permutation entropy
MSSE	Multi-scale sample entropy
MSSDE	Multi-scale symbolic dynamic entropy
MSRCSDFE	Multi-scale refined composite standard deviation fuzzy entropy
MHMSFDE	Multivariable hierarchical multi-scale fluctuation dispersion entropy
mRMR	Max-relevance min-redundancy
OR	Outer race
PCA	Principal component analysis
PE	Permutation entropy
PGB	Planetary gearboxes
PSO	Particle swarm optimization
PNN	Probabilistic neural network
RCFOA	Reverse cognitive fruit fly optimization algorithm
RCMSAE	Refined composite multi-scale approximate entropy
RCMSDE	Refined composite multi-scale dispersion entropy
RCMSFE	Refined composite multi-scale fuzzy entropy
RF	Random forest
SFE	Symbolic fuzzy entropy
SVM	Support vector machine
SWD	Swarm decomposition
SMCMSDE	Stacking modified composite multi-scale dispersion entropy
t-SNE	t-distributed stochastic neighbor embedding
TSMSWPE	Time-shift multi-scale weighted permutation entropy
TSMSDE	Time-shift multi-scale dispersion entropy
VPMCD	Variable predictive models based discrimination
WMSFDE	Weighted multi-scale fluctuation-based dispersion entropy
WRCMSDE	Weighted refined composite multi-scale dispersion entropy
WRCMSDE	Weighted refined composite multi-scale dispersion entropy
WRCMSFE	Weighted refined composite multi-scale fuzzy entropy
WRCMSPE	Weighted refined composite multi-scale permutation entropy

## References

[1] Shannon C., Weaver W. (1949). The Mathematical Theory of Communication.

[2] Chen W., Wang Z., Xie H., Yu W. (2007). Characterization of Surface EMG Signal Based on Fuzzy Entropy. IEEE Trans. Neural Syst. Rehabil. Eng..

[3] Richman J.S. (2007). Sample Entropy Statistics and Testing for Order in Complex Physiological Signals. Commun. Stat. Theory Methods.

[4] Sharma S., Tiwari S. (2022). A novel feature extraction method based on weighted multi-scale fluctuation based dispersion entropy and its application to the condition monitoring of rotary machines. Mech. Syst. Signal Process..

[5] Zhang X., Zhang M., Wan S., He Y., Wang X. (2021). A bearing fault diagnosis method based on multiscale dispersion entropy and GG clustering. Measurement.

[6] Martínez M., Guerra Carmenate J., Antonino-Daviu J., Dunai L., Fernández de Córdoba P., Velasco-Pla P., Conejero A. Spectral Entropy and Frequency Cepstral Coefficients of Stray Flux Signals for Sparking Detection in DC Motors. Proceedings of the 2023 IEEE 14th International Symposium on Diagnostics for Electrical Machines, Power Electronics and Drives.

[7] Sharma S., Tiwari S., Singh S. (2021). The rotary machine fault detection by hybrid method based on local mean decomposition and fluctuation based dispersion entropy. Mater. Today Proc..

[8] Minhas A., Singh S., Sharma N., Kankar P. (2020). Improvement in classification accuracy and computational speed in bearing fault diagnosis using multiscale fuzzy entropy. J. Braz. Soc. Mech. Sci. Eng..

[9] Rostaghi M., Azami H. (2016). Dispersion Entropy: A Measure for Time-Series Analysis. IEEE Signal Process. Lett..

[10] Li Y., Wang X., Liu Z., Liang X., Si S. (2018). The Entropy Algorithm and Its Variants in the Fault Diagnosis of Rotating Machinery: A Review. IEEE Access.

[11] Camarena-Martinez D., Valtierra-Rodriguez M., Amezquita-Sanchez J., Lieberman D., Romero-Troncoso R., Garcia-Perez A. (2016). Shannon Entropy and K -Means Method for Automatic Diagnosis of Broken Rotor Bars in Induction Motors Using Vibration Signals. Shock Vib..

[12] Eskov V., Eskov V., Vochmina Y.V., Gorbunov D., Ilyashenko L. (2017). Shannon entropy in the research on stationary regimes and the evolution of complexity. Mosc. Univ. Phys. Bull..

[13] Zenil H., Hernández-Orozco S., Kiani N.A., Soler-Toscano F., Rueda-Toicen A., Tegnér J. (2018). A Decomposition Method for Global Evaluation of Shannon Entropy and Local Estimations of Algorithmic Complexity. Entropy.

[14] De Queiroz M.M., Silva R.W., Loschi R.H. (2016). Shannon entropy and Kullback–Leibler divergence in multivariate log fundamental skew-normal and related distributions. Can. J. Stat..

[15] Bafroui H.H., Ohadi A. (2014). Application of wavelet energy and Shannon entropy for feature extraction in gearbox fault detection under varying speed conditions. Neurocomputing.

[16] Dong S., Xu X., Luo J. (2017). Mechanical Fault Diagnosis Method Based on LMD Shannon Entropy and Improved Fuzzy C-means Clustering. Int. J. Acoust. Vib..

[17] Li Y., Wang S., Yang Y., Deng Z. (2022). Multiscale symbolic fuzzy entropy: An entropy denoising method for weak feature extraction of rotating machinery. Mech. Syst. Signal Process..

[18] Sampaio D.L., Nicoletti R. (2016). Detection of cracks in shafts with the Approximated Entropy algorithm. Mech. Syst. Signal Process..

[19] Yentes J.M., Hunt N., Schmid K.K., Kaipust J.P., McGrath D., Stergiou N. (2013). The appropriate use of approximate entropy and sample entropy with short data sets. Ann. Biomed. Eng..

[20] Tomčala J. (2020). New fast ApEn and SampEn entropy algorithms implementation and their application to supercomputer power consumption. Entropy.

[21] Vega C.H.F., Noel J., Fernández J.R. Cognitive task discrimination using approximate entropy (ApEn) on EEG signals. Proceedings of the 2013 ISSNIP Biosignals and Biorobotics Conference: Biosignals and Robotics for Better and Safer Living (BRC).

[22] Alù F., Miraglia F., Orticoni A., Judica E., Cotelli M., Rossini P.M., Vecchio F. (2020). Approximate entropy of brain network in the study of hemispheric differences. Entropy.

[23] Chuckravanen D. (2014). Approximate entropy as a measure of cognitive fatigue: An eeg pilot study. Int. J. Emerg. Trends Sci. Technol..

[24] Lee G.M., Fattinger S., Mouthon A.L., Noirhomme Q., Huber R. (2013). Electroencephalogram approximate entropy influenced by both age and sleep. Front. Neuroinform..

[25] Yan R., Gao R. (2007). Approximate Entropy as a diagnostic tool for machine health monitoring. Mech. Syst. Signal Process..

[26] Zhao S., Liang L., Xu G., Wang J., Zhang W. (2013). Quantitative diagnosis of a spall-like fault of a rolling element bearing by empirical mode decomposition and the approximate entropy method. Mech. Syst. Signal Process..

[27] An X., Pan L. (2017). Wind turbine bearing fault diagnosis based on adaptive local iterative filtering and approximate entropy. Proc. Inst. Mech. Eng. Part C J. Mech. Eng. Sci..

[28] Ma Y., Cheng J., Wang P., Wang J., Yang Y. (2021). Rotating machinery fault diagnosis based on multivariate multiscale fuzzy distribution entropy and Fisher score. Measurement.

[29] Wu S.D., Wu C.W., Wu T.Y., Wang C.C. (2013). Multi-Scale Analysis Based Ball Bearing Defect Diagnostics Using Mahalanobis Distance and Support Vector Machine. Entropy.

[30] Ouyang G., Li J., Liu X., Li X. (2013). Dynamic characteristics of absence EEG recordings with multiscale permutation entropy analysis. Epilepsy Res..

[31] Yang Y., Zhou M., Niu Y., Li C., Cao R., Wang B., Yan P., Ma Y., Xiang J. (2018). Epileptic seizure prediction based on permutation entropy. Front. Comput. Neurosci..

[32] Ferlazzo E., Mammone N., Cianci V., Gasparini S., Gambardella A., Labate A., Latella M.A., Sofia V., Elia M., Morabito F.C. (2014). Permutation entropy of scalp EEG: A tool to investigate epilepsies: Suggestions from absence epilepsies. Clin. Neurophysiol..

[33] Yin Y., Shang P. (2014). Weighted multiscale permutation entropy of financial time series. Nonlinear Dyn..

[34] Henry M., Judge G. (2019). Permutation entropy and information recovery in nonlinear dynamic economic time series. Econometrics.

[35] He C., Wu T., Liu C., Chen T. (2020). A novel method of composite multiscale weighted permutation entropy and machine learning for fault complex system fault diagnosis. Measurement.

[36] Jinde Z., Junsheng C., Yang Y. (2014). Multiscale Permutation Entropy Based Rolling Bearing Fault Diagnosis. Shock Vib..

[37] Chen Y., Zhang T., Zhao W., Luo Z., Sun K. (2019). Fault Diagnosis of Rolling Bearing Using Multiscale Amplitude-Aware Permutation Entropy and Random Forest. Algorithms.

[38] Yasir M.N., Koh B.H. (2018). Data Decomposition Techniques with Multi-Scale Permutation Entropy Calculations for Bearing Fault Diagnosis. Sensors.

[39] Rajabi S., Saman Azari M., Santini S., Flammini F. (2022). Fault diagnosis in industrial rotating equipment based on permutation entropy, signal processing and multi-output neuro-fuzzy classifier. Expert Syst. Appl..

[40] Tiwari R., Gupta V., Kankar P. (2013). Bearing fault diagnosis based on multi-scale permutation entropy and adaptive neuro fuzzy classifier. J. Vib. Control.

[41] Xu F., Tse P. (2019). A method combining refined composite multiscale fuzzy entropy with PSO-SVM for roller bearing fault diagnosis. J. Cent. South Univ..

[42] Vakharia V., Gupta V., Kankar P. (2015). A multiscale permutation entropy based approach to select wavelet for fault diagnosis of ball bearings. J. Vib. Control.

[43] Li Y., Xu M., Wei Y., Huang W. (2016). A new rolling bearing fault diagnosis method based on multiscale permutation entropy and improved support vector machine based binary tree. Measurement.

[44] Zheng J., Pan H., Yang S., Cheng J. (2018). Generalized composite multiscale permutation entropy and Laplacian score based rolling bearing fault diagnosis. Mech. Syst. Signal Process..

[45] Kuai M., Cheng G., Pang Y., Li Y. (2018). Research of planetary gear fault diagnosis based on permutation entropy of CEEMDAN and ANFIS. Sensors.

[46] Du W., Guo X., Wang Z., Wang J., Yu M., Li C., Wang G., Wang L., Guo H., Zhou J. (2020). A New Fuzzy Logic Classifier Based on Multiscale Permutation Entropy and Its Application in Bearing Fault Diagnosis. Entropy.

[47] Zheng J., Dong Z., Pan H., Ni Q., Liu T., Zhang J. (2019). Composite multi-scale weighted permutation entropy and extreme learning machine based intelligent fault diagnosis for rolling bearing. Measurement.

[48] Xue X., Li C., Cao S., Sun J., Liu L. (2019). Fault Diagnosis of Rolling Element Bearings with a Two-Step Scheme Based on Permutation Entropy and Random Forests. Entropy.

[49] Dong Z., Zheng J., Huang S., Pan H., Liu Q. (2019). Time-Shift Multi-scale Weighted Permutation Entropy and GWO-SVM Based Fault Diagnosis Approach for Rolling Bearing. Entropy.

[50] Sharma S., Tiwari S., Singh S. (2021). Integrated approach based on flexible analytical wavelet transform and permutation entropy for fault detection in rotary machines. Measurement.

[51] He C., Wu T., Gu R., Jin Z., Ma R., Qu H. (2021). Rolling bearing fault diagnosis based on composite multiscale permutation entropy and reverse cognitive fruit fly optimization algorithm—Extreme learning machine. Measurement.

[52] Minhas A.S., Singh S. (2021). A new bearing fault diagnosis approach combining sensitive statistical features with improved multiscale permutation entropy method. Knowl.-Based Syst..

[53] Vashishtha G., Chauhan S., Singh M., Kumar R. (2021). Bearing defect identification by swarm decomposition considering permutation entropy measure and opposition-based slime mould algorithm. Measurement.

[54] Richman J.S., Lake D.E., Moorman J.R. (2004). Sample entropy. Methods in Enzymology.

[55] Jie X., Cao R., Li L. (2014). Emotion recognition based on the sample entropy of EEG. Bio-Med Mater. Eng..

[56] Aktaruzzaman M., Sassi R. (2014). Parametric estimation of sample entropy in heart rate variability analysis. Biomed. Signal Process. Control.

[57] Hu X., Jiang J., Cao D., Egardt B. (2015). Battery health prognosis for electric vehicles using sample entropy and sparse Bayesian predictive modeling. IEEE Trans. Ind. Electron..

[58] Mahajan R., Morshed B.I. (2014). Unsupervised eye blink artifact denoising of EEG data with modified multiscale sample entropy, kurtosis, and wavelet-ICA. IEEE J. Biomed. Health Inform..

[59] Xavier S.F.A., da Silva Jale J., Stosic T., dos Santos C.A.C., Singh V.P. (2019). An application of sample entropy to precipitation in Paraíba State, Brazil. Theor. Appl. Climatol..

[60] Han M., Pan J. (2015). A fault diagnosis method combined with LMD, sample entropy and energy ratio for roller bearings. Measurement.

[61] Ni Q., Feng K., Wang K., Yang B., Wang Y. (2017). A case study of sample entropy analysis to the fault detection of bearing in wind turbine. Case Stud. Eng. Fail. Anal..

[62] Li Y., Feng K., Liang X., Zuo M.J. (2019). A fault diagnosis method for planetary gearboxes under non-stationary working conditions using improved Vold-Kalman filter and multi-scale sample entropy. J. Sound Vib..

[63] Guan Z., Liao Z., Li K., Chen P. (2019). A precise diagnosis method of structural faults of rotating machinery based on combination of empirical mode decomposition, sample entropy, and deep belief network. Sensors.

[64] Wang Z., Yao L., Cai Y. (2020). Rolling bearing fault diagnosis using generalized refined composite multiscale sample entropy and optimized support vector machine. Measurement.

[65] Versaci M., Morabito F.C. (2021). Image edge detection: A new approach based on fuzzy entropy and fuzzy divergence. Int. J. Fuzzy Syst..

[66] Oliva D., Abd Elaziz M., Hinojosa S., Oliva D., Abd Elaziz M., Hinojosa S. (2019). Fuzzy entropy approaches for image segmentation. Metaheuristic Algorithms for Image Segmentation: Theory and Applications.

[67] Xiang J., Li C., Li H., Cao R., Wang B., Han X., Chen J. (2015). The detection of epileptic seizure signals based on fuzzy entropy. J. Neurosci. Methods.

[68] Cao Z., Lin C.T. (2017). Inherent fuzzy entropy for the improvement of EEG complexity evaluation. IEEE Trans. Fuzzy Syst..

[69] Liu C., Li K., Zhao L., Liu F., Zheng D., Liu C., Liu S. (2013). Analysis of heart rate variability using fuzzy measure entropy. Comput. Biol. Med..

[70] Azami H., Fernández A., Escudero J. (2017). Refined multiscale fuzzy entropy based on standard deviation for biomedical signal analysis. Med Biol. Eng. Comput..

[71] Joshi D., Kumar S. (2014). Intuitionistic fuzzy entropy and distance measure based TOPSIS method for multi-criteria decision making. Egypt. Inform. J..

[72] Song Y., Fu Q., Wang Y.F., Wang X. (2019). Divergence-based cross entropy and uncertainty measures of Atanassov’s intuitionistic fuzzy sets with their application in decision making. Appl. Soft Comput..

[73] Zheng J., Pan H., Cheng J. (2017). Rolling bearing fault detection and diagnosis based on composite multiscale fuzzy entropy and ensemble support vector machines. Mech. Syst. Signal Process..

[74] Wei Y., Li Y., Xu M., Huang W. (2019). Intelligent Fault Diagnosis of Rotating Machinery Using ICD and Generalized Composite Multi-Scale Fuzzy Entropy. IEEE Access.

[75] Li Y., Miao B., Zhang W., Chen P., Liu J., Jiang X. (2019). Refined composite multiscale fuzzy entropy: Localized defect detection of rolling element bearing. J. Mech. Sci. Technol..

[76] Zheng J., Cheng J., Yang Y., Luo S. (2014). A rolling bearing fault diagnosis method based on multi-scale fuzzy entropy and variable predictive model-based class discrimination. Mech. Mach. Theory.

[77] Gao S., Wang Q., Zhang Y. (2021). Rolling Bearing Fault Diagnosis Based on CEEMDAN and Refined Composite Multiscale Fuzzy Entropy. IEEE Trans. Instrum. Meas..

[78] Chen X., Cheng G., Li H., Zhang M. (2016). Diagnosing planetary gear faults using the fuzzy entropy of LMD and ANFIS. J. Mech. Sci. Technol..

[79] Zair M., Rahmoune C., Djamel B. (2018). Multi-fault diagnosis of rolling bearing using fuzzy entropy of empirical mode decomposition, principal component analysis, and SOM neural network. Proc. Inst. Mech. Eng. Part C J. Mech. Eng. Sci..

[80] Malhotra A., Singh Minhas A., Singh S., Zuo M.J., Kumar R., Kankar P.K. (2021). Bearing fault diagnosis based on flexible analytical wavelet transform and fuzzy entropy approach. Mater. Today Proc..

[81] Li Y., Xu M., Zhao H., Huang W. (2016). Hierarchical fuzzy entropy and improved support vector machine based binary tree approach for rolling bearing fault diagnosis. Mech. Mach. Theory.

[82] Vallejo M., Gallego C.J., Duque-Muñoz L., Delgado-Trejos E. (2018). Neuromuscular disease detection by neural networks and fuzzy entropy on time-frequency analysis of electromyography signals. Expert Syst..

[83] Tran M.Q., Elsisi M., Liu M.K. (2021). Effective feature selection with fuzzy entropy and similarity classifier for chatter vibration diagnosis. Measurement.

[84] Zhao H., Sun M., Deng W., Yang X. (2017). A New Feature Extraction Method Based on EEMD and Multi-Scale Fuzzy Entropy for Motor Bearing. Entropy.

[85] Deng W., Zhang S., Zhao H., Yang X. (2018). A Novel Fault Diagnosis Method Based on Integrating Empirical Wavelet Transform and Fuzzy Entropy for Motor Bearing. IEEE Access.

[86] Zheng J., Jiang Z., Pan H. (2018). Sigmoid-based refined composite multiscale fuzzy entropy and t-SNE based fault diagnosis approach for rolling bearing. Measurement.

[87] Minhas A.S., Singh G., Singh J., Kankar P., Singh S. (2020). A novel method to classify bearing faults by integrating standard deviation to refined composite multi-scale fuzzy entropy. Measurement.

[88] Chen X., Qi X., Wang Z., Cui C., Wu B., Yang Y. (2021). Fault diagnosis of rolling bearing using marine predators algorithm-based support vector machine and topology learning and out-of-sample embedding. Measurement.

[89] Xiao Y., Kang N., Hong Y., Zhang G. (2017). Misalignment Fault Diagnosis of DFWT Based on IEMD Energy Entropy and PSO-SVM. Entropy.

[90] Liu C., Zhu L., Ni C. (2018). Chatter detection in milling process based on VMD and energy entropy. Mech. Syst. Signal Process..

[91] Ali H.S., Chakravorty A., Kalayan J., de Visser S.P., Henchman R.H. (2021). Energy–entropy method using multiscale cell correlation to calculate binding free energies in the SAMPL8 host–guest challenge. J. Comput.-Aided Mol. Des..

[92] Portillo D., García Orden J., Romero I. (2017). Energy–entropy–momentum integration schemes for general discrete non-smooth dissipative problems in thermomechanics. Int. J. Numer. Methods Eng..

[93] Xiao Y., Hong Y., Chen X., Chen W. (2017). The Application of Dual-Tree Complex Wavelet Transform (DTCWT) Energy Entropy in Misalignment Fault Diagnosis of Doubly-Fed Wind Turbine (DFWT). Entropy.

[94] Yang Z., Kong C., Wang Y., Rong X., Wei L. (2021). Fault diagnosis of mine asynchronous motor based on MEEMD energy entropy and ANN. Comput. Electr. Eng..

[95] Pang B., Tang G., Zhou C., Tian T. (2018). Rotor Fault Diagnosis Based on Characteristic Frequency Band Energy Entropy and Support Vector Machine. Entropy.

[96] Gao S., Ren Y., Zhang Y., Li T. (2021). Fault diagnosis of rolling bearings based on improved energy entropy and fault location of triangulation of amplitude attenuation outer raceway. Measurement.

[97] Rostaghi M., Ashory M.R., Azami H. (2019). Application of dispersion entropy to status characterization of rotary machines. J. Sound Vib..

[98] Yan X., Jia M. (2019). Intelligent fault diagnosis of rotating machinery using improved multiscale dispersion entropy and mRMR feature selection. Knowl.-Based Syst..

[99] Azami H., da Silva L.E.V., Omoto A.C.M., Humeau-Heurtier A. (2019). Two-dimensional dispersion entropy: An information-theoretic method for irregularity analysis of images. Signal Process. Image Commun..

[100] Zhang W., Zhou J. (2019). A Comprehensive Fault Diagnosis Method for Rolling Bearings Based on Refined Composite Multiscale Dispersion Entropy and Fast Ensemble Empirical Mode Decomposition. Entropy.

[101] Minhas A.S., Kankar P., Kumar N., Singh S. (2021). Bearing fault detection and recognition methodology based on weighted multiscale entropy approach. Mech. Syst. Signal Process..

[102] Shao K., Fu W., Tan J., Wang K. (2021). Coordinated approach fusing time-shift multiscale dispersion entropy and vibrational Harris hawks optimization-based SVM for fault diagnosis of rolling bearing. Measurement.

[103] Tan H., Xie S., Liu R., Ma W. (2021). Bearing fault identification based on stacking modified composite multiscale dispersion entropy and optimised support vector machine. Measurement.

[104] Xue Q., Xu B., He C., Liu F., Ju B., Lu S., Liu Y. (2021). Feature Extraction Using Hierarchical Dispersion Entropy for Rolling Bearing Fault Diagnosis. IEEE Trans. Instrum. Meas..

[105] Zhou F., Han J., Yang X. (2021). Multivariate hierarchical multiscale fluctuation dispersion entropy: Applications to fault diagnosis of rotating machinery. Appl. Acoust..

[106] Wu S.D., Wu C.W., Lee K.Y., Lin S.G. (2013). Modified multiscale entropy for short-term time series analysis. Phys. A Stat. Mech. Its Appl..

[107] Wu S.D., Wu C.W., Lin S.G., Lee K.Y., Peng C.K. (2014). Analysis of complex time series using refined composite multiscale entropy. Phys. Lett. A.

[108] Miskovic V., MacDonald K.J., Rhodes L.J., Cote K.A. (2019). Changes in EEG multiscale entropy and power-law frequency scaling during the human sleep cycle. Hum. Brain Mapp..

[109] Yang A.C., Huang C.C., Yeh H.L., Liu M.E., Hong C.J., Tu P.C., Chen J.F., Huang N.E., Peng C.K., Lin C.P. (2013). Complexity of spontaneous BOLD activity in default mode network is correlated with cognitive function in normal male elderly: A multiscale entropy analysis. Neurobiol. Aging.

[110] Bhattacharyya A., Pachori R.B., Upadhyay A., Acharya U.R. (2017). Tunable-Q wavelet transform based multiscale entropy measure for automated classification of epileptic EEG signals. Appl. Sci..

[111] Silva L.E., Duque J.J., Felipe J.C., Murta Jr L.O., Humeau-Heurtier A. (2018). Two-dimensional multiscale entropy analysis: Applications to image texture evaluation. Signal Process..

[112] Agarwal A., Maheswaran R., Sehgal V., Khosa R., Sivakumar B., Bernhofer C. (2016). Hydrologic regionalization using wavelet-based multiscale entropy method. J. Hydrol..

[113] Zhou Y., Wang H., Wang G., Kumar A., Sun W., Xiang J. (2023). Semi-Supervised Multiscale Permutation Entropy-Enhanced Contrastive Learning for Fault Diagnosis of Rotating Machinery. IEEE Trans. Instrum. Meas..

[114] Lizarraga-Morales R.A., Rodriguez-Donate C., Cabal-Yepez E., Lopez-Ramirez M., Ledesma-Carrillo L.M., Ferrucho-Alvarez E.R. (2017). Novel FPGA-based methodology for early broken rotor bar detection and classification through homogeneity estimation. IEEE Trans. Instrum. Meas..

[115] Germán-Salló Z. (2020). Entropy indices based fault detection. Procedia Manuf..

[116] Dhandapani R., Mitiche I., McMeekin S., Morison G. Bearing Faults Diagnosis and Classification Using Generalized Gaussian Distribution Multiscale Dispersion Entropy Features. Proceedings of the 2022 30th European Signal Processing Conference (EUSIPCO).

[117] Chen J., Wen L., Jiang B., Lu N., Liu J. Multi-feature fusion and IGWO-LSSVM based fault diagnosis of rolling bearings. Proceedings of the 2023 CAA Symposium on Fault Detection, Supervision and Safety for Technical Processes (SAFEPROCESS).

[118] Ma C., Cai Z., Li Y., Wang X. Bearing Fault Detection Based on Multiresolution Permutation Entropy. Proceedings of the 2023 5th International Conference on System Reliability and Safety Engineering (SRSE).

